# Bacterial Biodiversity-Ecosystem Functioning Relations Are Modified by Environmental Complexity

**DOI:** 10.1371/journal.pone.0010834

**Published:** 2010-05-26

**Authors:** Silke Langenheder, Mark T. Bulling, Martin Solan, James I. Prosser

**Affiliations:** 1 Institute of Biological and Environmental Sciences, University of Aberdeen, Aberdeen, United Kingdom; 2 Oceanlab, University of Aberdeen, Newburgh, United Kingdom; University of Oxford, United Kingdom

## Abstract

**Background:**

With the recognition that environmental change resulting from anthropogenic activities is causing a global decline in biodiversity, much attention has been devoted to understanding how changes in biodiversity may alter levels of ecosystem functioning. Although environmental complexity has long been recognised as a major driving force in evolutionary processes, it has only recently been incorporated into biodiversity-ecosystem functioning investigations. Environmental complexity is expected to strengthen the positive effect of species richness on ecosystem functioning, mainly because it leads to stronger complementarity effects, such as resource partitioning and facilitative interactions among species when the number of available resource increases.

**Methodology/Principal Findings:**

Here we implemented an experiment to test the combined effect of species richness and environmental complexity, more specifically, resource richness on ecosystem functioning over time. We show, using all possible combinations of species within a bacterial community consisting of six species, and all possible combinations of three substrates, that diversity-functioning (metabolic activity) relationships change over time from linear to saturated. This was probably caused by a combination of limited complementarity effects and negative interactions among competing species as the experiment progressed. Even though species richness and resource richness both enhanced ecosystem functioning, they did so independently from each other. Instead there were complex interactions between particular species and substrate combinations.

**Conclusions/Significance:**

Our study shows clearly that both species richness and environmental complexity increase ecosystem functioning. The finding that there was no direct interaction between these two factors, but that instead rather complex interactions between combinations of certain species and resources underlie positive biodiversity ecosystem functioning relationships, suggests that detailed knowledge of how individual species interact with complex natural environments will be required in order to make reliable predictions about how altered levels of biodiversity will most likely affect ecosystem functioning.

## Introduction

The ability of ecological systems to continue to deliver the ecosystem services on which human well-being ultimately depends is being increasingly compromised by anthropogenic endeavour, including pressures resulting from environmental change and invasion of exotic species [1]–[2]. Such pressures have also led to a rapid decline in biodiversity on a global scale, and gaining an understanding of the relationships between biodiversity and ecosystem functioning has been the primary objective of a substantial amount of literature over the last 10–15 years. Early experiments on plant communities showed declines in biomass production resulting from declines in species richness [3]–[4], and triggered a large number of equivalent studies in other systems. When these data are integrated [5]–[7], the relationships between biodiversity and ecosystem functioning (BEF) are mostly positive, but the most species-rich mixture tends to generate a level of functioning that is no different from that of the single most productive species used in an experiment [6], suggesting that a limited number of species rather than diversity *per se* determine the shape of the curve [8].

However, studies investigating BEF relationships in the presence of abiotic and biotic external drivers [9]–[13] and their variation over time [14] are scarce. Determination of the quantitative relationships between species richness and levels of ecosystem functioning is severely inhibited by an inability to study communities in sufficient numbers and with the control, replication and realism required [15]. BEF experiments involving microbial communities offer great potential for addressing these issues, because they are relatively easily manipulated and conditions can be readily replicated in the laboratory. Moreover, given the overwhelming importance of bacterial assemblages and processes for ecosystem sustainability and overall functioning, and the desire to test the universality of BEF relationships observed in plant and animal communities, knowledge of BEF relationships for bacterial assemblages is urgently required. Recent studies investigating BEF relationships in bacteria [16]–[17] generally confirm the trend of a positive effect of species richness on ecosystem processes, but there is also evidence of saturation at low species richness levels when redundant, i.e. functionally equivalent species are present [18], and negative sampling effects, where species with low contributions to ecosystem functioning dominate in species-rich communities [19].

Resource limitation can affect the behaviour and competitive ability of species, and environmental complexity in the form of multiple resources can sustain greater biodiversity by reducing interspecific competition through greater differentiation of resource use between species [20]. However, the interactive effects of environmental complexity and species diversity on ecosystem functioning have only recently been examined [12], [21]–[23]. It can be hypothesised that ecosystem functioning increases with both higher species and resource richness due to stronger complementarity effects. Firstly, higher species and resource richness may reduce competition among species due to resource differentiation and, secondly, they may enhance facilitative interactions, such as cross-feeding on metabolic by-products among species. Previous studies have found evidence for [e.g. 22] and against [e.g. 24] stronger effects of increasing species richness on ecosystem functioning with increasing resource heterogeneity. Moreover, environmental heterogeneity may influence sampling effects that underpin positive BEF relationships. Resource heterogeneity may, for example, lower sampling effects by reducing the impact of a dominant species on ecosystem functioning when the presence of several resources leads to a reduction in competition. One can hypothesise that this in itself, if not counteracted or compensated by increasing complementary effects, may lead to a negative effect of resource richness on BEF relationships, unless the same species dominates irrespective of the degree of resource heterogeneity [24].

In our study we make use of the significant benefits that microorganisms offer as model systems to test and inform ecological theory [25]–[26] to examine whether interaction effects between bacterial species richness and environmental complexity (resource richness) determine levels of ecosystem functioning over time. We included all possible species combinations per species richness level (n = 6), six sampling times and all combinations within each resource richness level (n = 3, using glucose, xylose and galactose as substrates). The major emphasis of this study is to test how ecosystem functioning changes over time, depending on species richness and substrate richness, utilising a full experimental design incorporating all possible species and substrate combinations, whilst ensuring the validity of our findings by avoiding any undersampling biases related to missing species or substrate combinations. We hypothesise that substrate richness will enhance the positive effects of species richness on ecosystem functioning.

## Results

### Effects of species richness, species composition and substrate richness on metabolic activity over time

**Figure 1:**
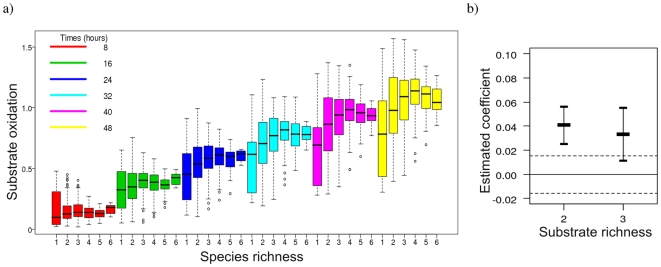
Influence of species and resource richness on the metabolic activity of bacterial communities. Metabolic activity (substrate oxidation) was determined as colour development, measured spectrophotometrically at 600 nm, resulting from reduction of a redox dye (tetrazolium violet) added to the culture medium. (**a**) A boxplot of metabolic activity (substrate oxidation) over time at each level of species richness (1–6). Boundaries of boxes indicate the interquartile range (25^th^ to 75^th^ percentiles) and median value (midline); whiskers indicate 1.5 times the interquartile range. Individual points (open circles) indicate outliers. (**b**) Estimation of coefficient values for the significant single term substrate richness obtained using Restricted Maximum Likelihood (REML) estimation. The coefficients represent effects for species richness levels of 2 and 3 relative to a baseline level at a species richness of 1. Error bars represent 95% confidence intervals.

For the species richness model two terms, species richness×time and substrate richness, significantly explained metabolic activity (Table 1). Metabolic activity of communities consistently increased with time irrespective of species richness level (Fig. 1a), reflecting cumulative activity during the course of the experiment. Species richness had a positive effect on activity that increased during incubation (Fig. 1a), but curves showed saturation at later times. Moreover, variance in metabolic activity increased with time, but decreased with richness (Fig. 1a). Activity was greater in communities supplied with multiple rather than single substrates, across all species richness levels (Table 1, Fig. 1b), and was also influenced by substrate composition, i.e. the identity of substrates in the medium (Table 1). Species composition also had a significant effect on metabolic activity, in combination with substrate richness and time (Table 1). Activities of the six monocultures differed several-fold, with species B having greatest activity (Fig. S1). Communities containing species B generally showed higher metabolic activity at each species richness level and this effect increased with incubation time (compare panels a–c, Fig. S1).

**Table 1:** Summary of linear regression models to analyse 1. metabolic activity, 2. non-transgressive overyielding, and 3. transgressive overyielding.

Independent variables	Significant terms	L	d.f.	p-value
***1) Metabolic activity analysis***				
SR, SUBR, T	SR×T	132,4	30	<0.0001
	SUBR	44.83	12	<0.0001
SC, SUBR, T	SC×T×SUBR	742.85	620	<0.001
***2) Non-transgressive overyielding analysis***				
SR, SUBR, T	SR×T	94.65	20	<0.0001
	SUBR×T	31.14	10	<0.0001
SR, SUBC, T	SR×T	103.86	20	<0.0001
	SUBC×T	128.91	30	<0.0001
***3) Transgressive overyielding analysis***				
SR, SUBR, T	SR	280.53	4	<0.01
	SUBR×T	26.08	10	<0.01
SR, SUBC, T	SR×SUBC	37.23	23	<0.0001
	SUBC×T	160.99	30	<0.0001

The models were designed to investigate effects of species richness, species composition and substrate richness on metabolic activity, and species richness, substrate richness and substrate composition on non-transgressive and transgressive overyielding over time, respectively. SR: species richness, T: time, SUBR: substrate richness, SC: species composition, SUBC: substrate composition. L: L-value, d.f.: degrees of freedom.

### Analysis of non-transgressive and transgressive overyielding

**Figure 2:**
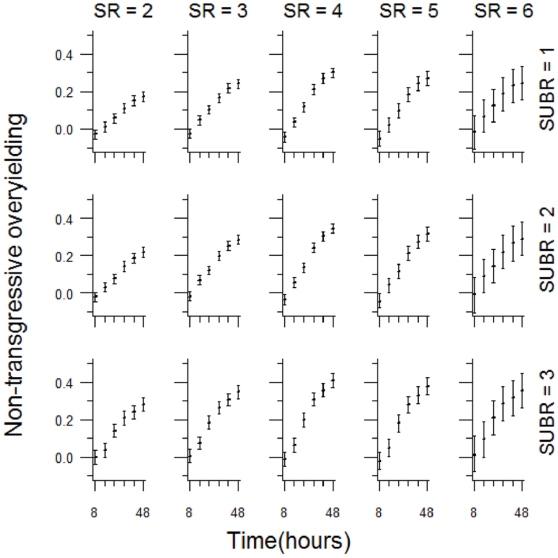
Visualisation of the non-transgressive overyielding model incorporating substrate richness, species richness and time as independent variables. Predictions from the model illustrating the significant 2-way interactions time×species richness and time×substrate richness. Although the effects of the 2-way interaction terms are independent of each other, the independent variable time is involved in both. For clarity we therefore show model predictions over the full range of all three independent variables, incorporating significant interaction effects simultaneously rather than in two separate figures. Confidence intervals (95%) were estimated using a bootstrapping procedure (see Methods). Columns represent distinct species richness (SR) levels and rows represent distinct substrate richness (SUBR) levels.

In general, non-transgressive overyielding was observed, i.e. species combinations often achieved higher yields than would have been expected from the respective mean monoculture performances (Fig. 2, Fig. S2). The statistical model incorporating substrate richness contained two significant 2-way interaction terms, species richness***×***time and substrate richness***×***time (Table 1). Non-transgressive overyielding increased with time but this increase was modified by the interaction with species richness. The lowest levels of non-transgressive overyielding were associated with the lowest species richness ( = 2) (Fig. 2). During the early stages of incubation non-transgressive overyielding was greatest at the highest richness level. However, towards the later stages of incubation, the intermediate richness levels (i.e. 4 and 5) generated the highest levels of non-transgressive overyielding. Non-transgressive overyielding was also positively associated with greater substrate richness (Fig. 2).

**Figure 3:**
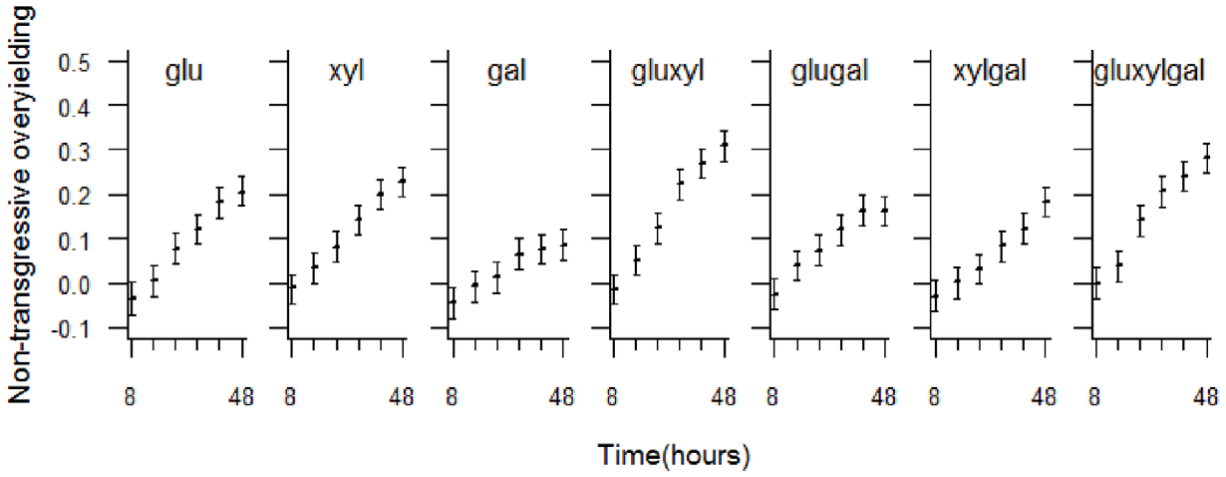
Visualisation of the non-transgressive overyielding model with substrate composition, species richness and time as independent variables. Predictions from the model illustrating the significant 2-way interaction time×substrate composition. Confidence intervals (95%) were estimated using a bootstrapping procedure (see Methods). gal = galactose, glu = glucose, xyl = xylose, with pairs of these representing a substrate richness level of 2 and the triplet representing a substrate richness of 3.

The statistical model incorporating substrate composition rather than substrate richness had a similar structure with the two significant 2-way interaction terms, species richness***×***time and substrate composition***×***time (Table 1). There were clear substrate composition effects, and this analysis revealed additional complexity to the general trend of increasing substrate richness, which tended to be associated with greater levels of non-transgressive overyielding (Fig. 3).

**Figure 4:**
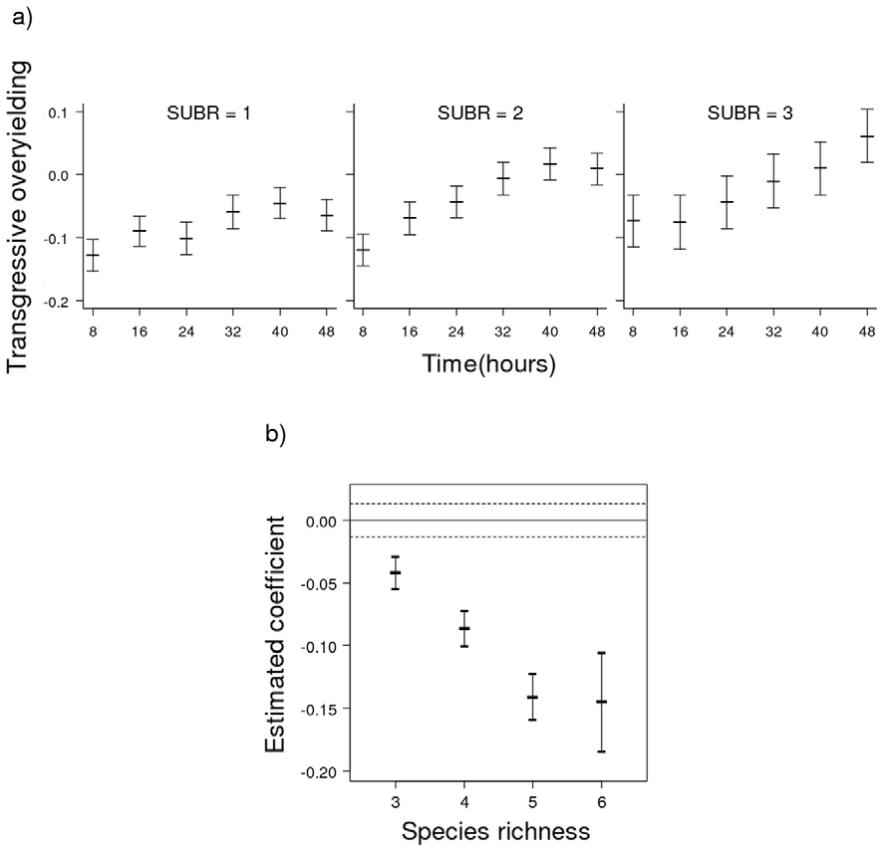
Visualisation of the transgressive overyielding model with substrate richness, species richness and time as independent variables. Predictions from the model illustrating (**a**) the significant 2-way interaction time×substrate richness (SUBR). Confidence intervals (95%) were estimated using a bootstrapping procedure (see Methods); (**b**) the estimates of coefficient values for the significant single term variable species richness obtained using Restricted Maximum Likelihood (REML) estimation. The coefficients represent effects for species richness levels of 3, 4, 5 and 6 relative to a baseline level at a species richness of 2. Error bars represent 95% confidence intervals.

**Figure 5:**
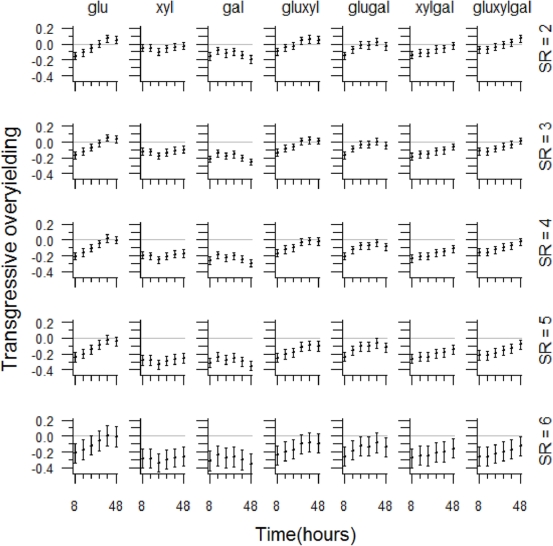
Visualisation of the transgressive overyielding model with substrate composition, species richness and time as independent variables. Predictions from the model illustrating the significant 2-way interaction terms: time×substrate composition and species richness×substrate composition. Although the effects of the 2-way interaction terms are independent of each other, the independent variable substrate composition is involved in both. For clarity we therefore show model predictions over the full range of all three independent variables, incorporating significant interaction effects simultaneously rather than in two separate figures. Confidence intervals (95%) were estimated using a bootstrapping procedure (see Methods). Columns represent distinct substrate composition levels and rows represent distinct species richness (SR) levels. gal = galactose, glu = glucose, xyl = xylose, with pairs of these representing a substrate richness level of 2 and the triplet representing a substrate richness of 3. The horizontal grey line in each plot is a visual reference indicating the level of zero transgressive overyielding.

In contrast to non-transgressive overyielding, there was generally no transgressive overyielding (Figs. 4 and 5, Fig. S2), i.e. species combinations generally produced lower yields than the maximum respective monocultures. The statistical model incorporating substrate richness did not contain a significant interaction between species richness and time, but did include the significant 2-way interaction substrate richness***×***time as well as a single-term effect with species richness (Table 1). Species mixtures came closer to transgressive overyielding with time and this trend was greater with the two higher substrate richness levels (Fig. 4a). Higher species richness levels were associated with yields that were further from transgressive overyielding, although there was no difference between the regression coefficients for the two highest species richness levels (5 and 6) (Fig. 4b). The statistical model incorporating substrate composition rather than substrate richness had a more complex structure, including the two 2-way interaction terms, species richness***×***substrate composition and substrate composition***×***time (Table 1). This model revealed strong substrate composition effects on transgressive overyielding and levels of complexity not shown in the equivalent model utilising substrate richness (Fig. 5). The single substrate treatments xylose and galactose were associated with cases furthest from those showing transgressive overyielding, driving part of the substrate richness effect in the previous model. In contrast, the single substrate treatment glucose was associated with less negative values, including transgressive overyielding at the latest times. The 2- and 3 substrate treatments generally produced mid- to high range levels. There was also a noticeable decline in levels of transgressive overyielding at the later times associated with the single substrate treatments galactose and glucose.

## Discussion

### Mechanisms underpinning bacterial diversity-ecosystem functioning relations

A positive relationship was observed between species richness and metabolic activity. However, the form of this relationship changed with time, initially tending towards a linear relationship and, at later times, exhibiting saturation. Previous experiments linking bacterial diversity to decomposition have also found saturating relationships between diversity and functioning [18]–[19]. The underlying mechanisms were presumed to be complementarity in the first study [18] and negative sampling effects in the second [19], where communities were dominated by a species that did not contribute strongly to decomposition.

Generally, both complementarity and sampling effects have been proposed to explain positive diversity-functioning relationships [27]–[29] and the available data suggest that, in most cases, a combination of the two is likely to be operating [14], [30]. Complementarity refers to increased resource utilisation through partitioning or positive interaction, whereas the sampling effect describes the influence of dominance by species with particular traits on ecosystem processes. In the latter case, the variation in dominance could result from changes in the densities of species (i.e. population dynamics) and/or differences in the rates at which species contribute to ecosystem functioning. The experimental design adopted here minimised changes in relative abundance of each species with time, but did not remove the potential for sampling effects through differences in metabolic rates among species, as substrates were utilised and end-products accumulated. Thus, in this experiment, we cannot rule out that these sampling effects could have masked complementarity effects.

Non-transgressive overyielding was observed with all substrates at times later than 8 hours, but this extended to transgressive overyielding in far fewer cases. Hence, species mixtures tended to have higher yields than the average monoculture yield, but exceeded the maximum monoculture yield to a much lower extent. Similarly, Cardinale et al. [14] observed that plant communities tend to show non-transgressive but not transgressive overyielding and that both expressions of overyielding increase with time. The rare occurrence of transgressive overyielding suggests that true complementarity effects were not common in our experiment. Consequently, the saturation of functioning could have been caused by the lack of complementarity effects at higher species richness levels. Alternatively, negative interactions among competing species might explain the saturation. The system had a high level of functional redundancy, with all species able to utilise all supplied substrates and there was, therefore, presumably limited scope for positive complementarity effects, such as niche differentiation and facilitation. Thus, it seems more likely that negative interactions, due to increased competition between species for a limited amount and number of substrates in more diverse communities, could have led to saturation in levels of ecosystem functioning. This is supported by the hypothesis that strong niche differences between co-existing species are required for transgressive overyielding [31].

### Interactive effects of resource heterogeneity and species diversity

Environmental heterogeneity includes the richness of the available substrate pool. In bacterial populations, it has been shown that substrate diversity fosters diversification via adaptive radiation [32]–[33], increases in specific growth rates of single bacterial strains [e.g. 34] and changes in composition and functioning of natural microbial communities [e.g. 35]. However, the interactive effects of substrate richness and species richness on EF are much less explored [but see 22]. Here, a positive relationship was observed with increasing substrate richness for both non-transgressive and transgressive overyielding as well as metabolic activity. A positive relationship may reflect a change in species behaviour between substrate richness levels, with species altering their pattern of resource uptake in relation to the number of resources available and other species that are present. Such mechanisms should lead to stronger complementarity effects with increasing resource richness and result in higher metabolic activity. It was therefore surprising that a significant interaction between species richness and substrate richness was not observed in any of the models. Hence, our hypothesis that substrate richness would enhance positive effects of species richness on ecosystem functioning must be rejected. Even though species richness and resource richness both enhanced metabolic activity, non-transgressive and transgressive overyielding, they did so independently from each other. One possible cause for the lack of an interaction between species and substrate richness might be, as already mentioned earlier, the presumably limited scope for complementarity due to the high levels of functional redundancy in our model system. Moreover, facilitative interactions due to, for example, cross-feeding, might have been of minor importance due to the nature of the supplied substrates, which were probably completely degraded and therefore unlikely to have produced metabolic by-products that could be utilised by other species.

Instead of a direct interaction between species richness and substrate richness there were interactions between particular species and substrate combinations. This became apparent in the higher resolution model of transgressive overyielding that incorporated substrate composition as an independent variable, as it contained the significant 2-way interaction, species richness × substrate composition. Also the model of metabolic activity incorporating species composition contained the significant 3-way interaction term, species composition × substrate richness × time. Our results therefore suggest that the pattern of resource availability will be an important component in determining BEF relationships, but that the particular characteristics of the species and resources available are more important than the mere number of both. This further suggests that the effect of habitat changes in conjunction with species extinction on functioning will be difficult to predict, and general patterns within this interaction elusive.

Other studies with a focus on microbial communities have also investigated the effect of resource heterogeneity on BEF relationships. Replansky and Bell [22], in a long-term experiment, showed that both environmental complexity (i.e. the number of carbon substrates) and species richness tended to increase productivity of yeast communities, and that overyielding was mostly caused by resource partitioning. Tiunov and Scheu [36] investigated the effect of fungal species richness on decomposition and found stronger complementarity effects in less complex environments, where facilitative interactions between species, such as cross-feeding, were more prominent. Weis et al. [24], on the contrary, found that environmental heterogeneity did not have positive effects on BEF relationship in algal communities. However, a single species dominated in both homogeneous and heterogeneous environments in their experiment, hence, limiting the scope for complementarity effects. Thus, even though the overall effect and underlying mechanisms differ between different studies, probably depending on the overall heterogeneity in the studied system as well as whether specialists or generalists are competing for the available resources [37]–[38], the results of experiments carried out with microbial communities confirm the importance of habitat complexity reported elsewhere with larger organisms [e.g. 21], [39]–[41].

In summary, the experimental system here demonstrates that habitat complexity in the form of resource richness can be a significant modifying force of the BEF relationship. Predicting and managing changes in ecosystem functioning resulting from declining biodiversity is likely to require an extensive knowledge of these complex interactions, and as such, resource complexity must form a major component of future research into the relationship between biodiversity and ecosystem functioning.

## Methods

### Experimental design

Six bacterial species (*Rhodoferax* sp. strain SL-68, *Flavobacterium* sp. strain SL-104, *Sphingoterrabacterium* sp. strain SL-106, *Burkholderia* sp. strain SL-187, *Sphingobium yanoikuyae*, strain SL-197 and *Bacteroidetes* sp. strain SL-WC2) were isolated from Scottish soil (Text S1). Throughout the manuscript we use the following designation for the different strains: Species A (SL-68), species B (SL-104), species C (SL-106), species D (SL-187), species E (SL-197) and species F (SL-WC2). All possible combinations (n = 7) of 3 carbon resources (glucose, xylose and galactose), each of which could be utilised by each bacterial species, were assembled. Total resource concentration was fixed at 5.5 mM, irrespective of the number of component resources. The influence of species richness and species composition on total substrate utilisation was investigated by determining the metabolic activity of each combination of bacterial species (n = 63) on each substrate combination (n = 7) in microtitre plates (220 µl well^−1^, Nunclon TM delta surface, Nunc) without replication, resulting in a total of 441 cultures. Colour development, resulting from reduction of a redox dye (tetrazolium violet) added to the culture medium (Text S1), was used as a surrogate of metabolic activity, and was measured spectrophotometrically (λ = 600 nm) at 0, 8, 16, 24, 32, 40 and 48 h using a Thermomax microtitre plate reader (Molecular Devices, Wokingham, UK). The positions of all species richness-substrate combinations on six microplates were randomly assigned except that wells of the same column always contained the same substrate to facilitate inoculation of plates using a multi-channel pipette. Inoculation of all six microplates was achieved within 45 minutes. Controls were included consisting of medium without cells, but with substrate, to check for potential external contamination, and medium without substrate, but with cells, to account for any background resource utilisation. Absorbance values from plate readings were corrected for initial values prior to statistical analysis.

Inocula were prepared by growing strains from glycerol stock cultures to stationary phase on medium containing all 3 carbon sources. Cells were concentrated by centrifugation of 30–50 ml of cell suspension for 10 min at 12800×g in 50 ml Falcon tubes. Cells were washed by re-suspension of pellets in 7 ml of mineral salts medium (Text S1) and further diluted to the required cell concentration prior to inoculation of 200 µl mineral salts medium, supplemented with the required combination of substrates, with 20 µl of diluted cell suspension. Cell concentrations were estimated prior to inoculation of microtitre plates using previously established curves relating optical density at 600 nm (OD_600_) to cell concentration determined using epifluorescence microscopy after 4′-6-Diamidino-2-phenylindole- (DAPI-) staining. Final total cell concentration in the wells was ∼2×10^8^ cell ml^−1^, regardless of the number of component strains. Such high cell densities are close to the carrying capacity of the growth medium and were chosen to minimise growth effects and to focus attention on metabolic activity.

### Statistical analyses

#### Effects of species richness, species composition and substrate richness on metabolic activity over time

Two linear regression models (Text S2) were developed for the dependent variable (metabolic activity). In the first model, we used the independent nominal variables species richness (SR), substrate richness (SUBR) and time (T) to assess the effect of species and substrate richness on total metabolic activity. The second model examined the role of particular species combinations (SC), incorporating the independent variables species composition (the particular combination of species used), substrate richness and time. As measurements were taken from each well through time, we used a repeated measures structure, determining the most appropriate covariance structure (unstructured and allowing for different variances at each time point) using the Akaike Information Criteria (AIC) and utilising Restricted Maximum Likelihood (REML) estimation. Diagnostic plots of residuals versus fitted values revealed no significant heterogeneity of variance and QQ-plots indicated that assumptions of normality were justified. The fixed structure of the model was established by applying backward selection using the likelihood ratio test obtained by Maximum Likelihood (ML). The numerical output of the minimal adequate model was obtained using REML estimation [42]. All analyses were performed using the ‘nlme’ package (v 3.1, Pinheiro et al. [43]) in the ‘R’ statistical and programming environment.

#### Non-transgressive overyielding

To investigate potential mechanisms underlying the diversity-metabolic activity curve we determined the extent of non-transgressive overyielding, i.e. whether mixed communities perform better than would be expected from purely additive effects predicted from the performance of individual strains in monocultures. Thus, the non-transgressive overyield measurement of a particular community is the measured level of metabolic activity, minus the mean levels obtained from the monocultures of the constituent species. A positive result indicates non-transgressive overyielding.

Two statistical models were developed for the dependent variable non-transgressive overyielding. The first incorporated species richness (SR), substrate richness (SUBR) and time (T) to assess the effect of species and substrate richness on non-transgressive overyielding. The second examined the role of specific substrate combinations, incorporating the independent variables species richness, substrate combination and time. As each yield measurement was calculated relative to the mean monoculture levels of metabolic activity at the appropriate point in time, there was no requirement to use a repeated measures structure. However, severe heterogeneity of variance in the initial linear regression models required the application of a generalised least squares extension to determine appropriate variance-covariate terms and meet the assumptions of homogeneity of variance [[42], [44], see Text S3]. The appropriate variance-covariate structure was determined using AIC scores in conjunction with residual plots for model developed using REML. The model was then refined by manual backwards stepwise regression using ML to remove insignificant terms. The final model was presented using REML.

#### Transgressive Overyielding

Levels of transgressive overyielding [see 45 for details], using the appropriate maximum monoculture level of ecosystem functioning as the basis for comparison, were also calculated and analysed using the statistical framework used for non-transgressive overyielding. Positive transgressive overyielding unequivocally indicates positive effects of biodiversity [6], [14], [46].

#### Estimation of confidence intervals around model predictions

A bootstrap-routine was implemented for each model to obtain estimates of the 95% confidence intervals around model predictions [47]–[48]. This involved repeatedly (n = 2000) randomly reordering the model residuals, adding these to the fitted values from the original model, reapplying the model, and obtaining model predictions from the updated model. This resulted in distributions associated with every possible combination of levels of the independent variables from which estimated 95% confidence intervals could be obtained.

## Supporting Information

Figure S1 Interactive effects of bacterial species composition, resource richness and time on metabolic activity. Predicted levels of metabolic activity (substrate oxidation) for each of the 63 communities constructed from a 6 species pool, at 3 substrate richness levels at (a) 8, (b) 16 and (c) 48 h. Horizontal bars represent predicted values from the minimal adequate model for each level of resource richness. Species represented: *Rhodoferax* sp. SL-68, A; *Flavobacterium* sp. SL-104, B; *Sphingoterrabacterium* sp. SL-106, C; *Burkholderia* sp. SL-187, D; *Sphingobium yanoikuyae* SL-197, E; *Bacteroidetes* sp. SL-WC2, F. Mixtures are denoted by letter groupings.
(5.10 MB TIF)

Figure S2 Levels of non-transgressive overyielding and transgressive overyielding for each species combination on each substrate combination at 48 hours. Non-transgressive overyielding is labelled as “yield” and transgressive overyielding as “transgressive yield” in all plots. The codes for the substrates are glu = glucose, gal = galactose, xyl = xylose. Codes for species are as in Figure S1.
(0.24 MB DOC)

Text S1 Isolation of strains and preparation of communities. A detailed summary of the general procedure, media and PCR-protocols used during the isolation and characterisation of the six bacterial strains used in the experiment.
(0.03 MB DOC)

Text S2 Model details. Details of the linear regression models to analyse effects of species richness, species composition and substrate richness on 1) metabolic activity, 2) non-transgressive overyielding, and 3) transgressive overyielding.
(0.03 MB DOC)

Text S3 Description of GLS extension. Brief explanation of the generalised least squares (GLS) extension for linear regression models.
(0.03 MB DOC)
