# Supplementary figures and images for: Bacterial Biodiversity-Ecosystem Functioning Relations Are Modified by Environmental Complexity
Source: PLoS One. 2010 May 26;5(5):e10834. doi: 10.1371/journal.pone.0010834 (PMC2877076; doi:10.1371/journal.pone.0010834)

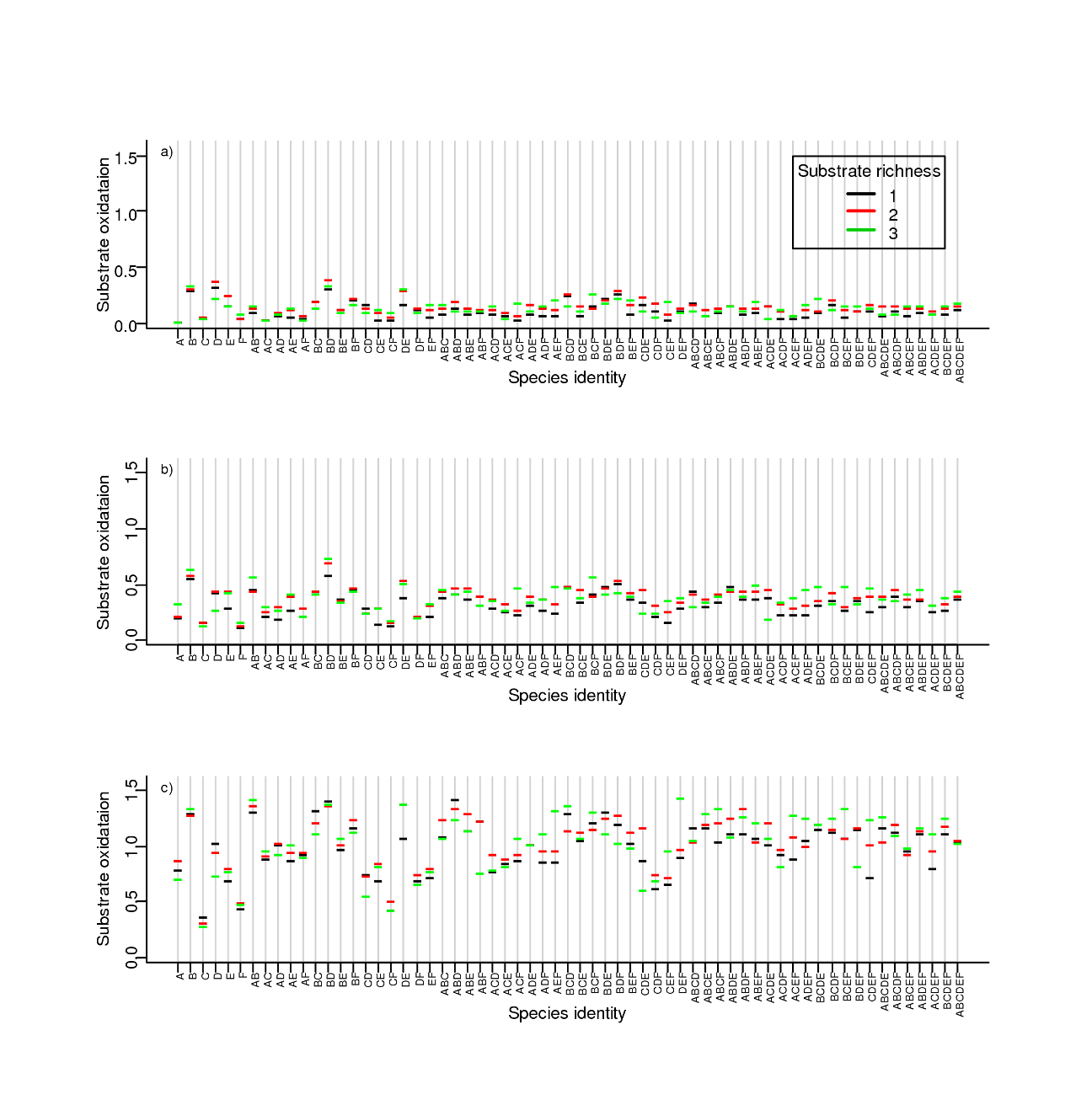

Supplement: Figure S1 — Interactive effects of bacterial species composition, resource richness and time on metabolic activity. Predicted levels of metabolic activity (substrate oxidation) for each of the 63 communities constructed from a 6 species pool, at 3 substrate richness levels at (a) 8, (b) 16 and (c) 48 h. Horizontal bars represent predicted values from the minimal adequate model for each level of resource richness. Species represented: Rhodoferax sp. SL-68, A; Flavobacterium sp. SL-104, B; Sphingoterrabacterium sp. SL-106, C; Burkholderia sp. SL-187, D; Sphingobium yanoikuyae SL-197, E; Bacteroidetes sp. SL-WC2, F. Mixtures are denoted by letter groupings. (5.10 MB TIF) [file pone.0010834.s001.tif]
