# Supplementary material for: Bacterial Biodiversity-Ecosystem Functioning Relations Are Modified by Environmental Complexity
Source: PLoS One. 2010 May 26;5(5):e10834. doi: 10.1371/journal.pone.0010834 (PMC2877076; doi:10.1371/journal.pone.0010834)
Supplement: Text S1 — Isolation of strains and preparation of communities. A detailed summary of the general procedure, media and PCR-protocols used during the isolation and characterisation of the six bacterial strains used in the experiment. (0.03 MB DOC) [file pone.0010834.s003.doc]

**Isolation of strains**

Bacterial strains were isolated from surface soil samples obtained from either garden soil, obtained from the Botanical Garden of the University of Aberdeen, or soil from an agricultural field (Scottish Agricultural College, Craibstone, Aberdeen, Scotland, Grid reference NJ872104). Dilutions of soil suspensions were plated onto half-strength mineral salts medium 457 (Brunner, Deutsche Sammlung von Mikroorganismen und Zellkulturen (DSMZ) Braunschweig, Germany) containing 0.5 mM glucose as the only carbon source, and solidified using 15 g L^‑1^ pure agar (Oxoid). Full-strength DSM 457 medium consists of: 2.44 g L^-1^ Na_2_HPO_4_, 1.52 g L^-1^ KH_2_PO_4_, 0.50 g L^-1^ (NH_4_)_2_SO_4_, g L^-1^ MgSO_4._7H_2_O, 0.05 g L^-1^ CaCl_2_.2H_2_O and 2 ml of trace element solution of the following composition: 0.5 g L^-1^ EDTA, 0.2 g L^-1^ FeSO_4_.7H_2_O, 0.01 g L^-1^ ZnSO_4_.7H_2_O, 0.003 g L^-1^ MnCl_2_.4H_2_O, 0.03 g L^-1^ H_3_BO_3_, 0.02 g L^-1^ CoCl_2_.6H_2_O, 0.001 gL^-1^ CuCl_2_.2H_2_O, 0.002 g L^-1^ NiCl.6H_2_O and 0.003 g L^-1^ Na_2_MoO_4_.2H_2_O. Plates were incubated at room temperature and, after 7 days, selected colonies were purified by repeated subculture on fresh agar plates and with gradual, successive increases in glucose concentration up to 2 mM. Isolates were then cultivated in the liquid mineral salts medium and DNA was extracted from fully grown cultures by adding 50 µl of a 5% Chelex-100 (Bio-Rad) solution to 1 ml of fully grown culture following centrifugation for 20 min at 10,000 rpm. Cells were lysed by subjecting the suspension to two cycles of incubation for 10 min at 100°C and immediate freezing at -20°C, followed by centrifugation for 20 min at 4,000 rpm. The supernatant was used for PCR amplification of 16S rRNA gene sequences using primers 27f and 1492r [1], targeting all bacteria, using the following conditions: 95°C for 3 min, followed by 25 cycles at 95°C for 1 min, 50°C for 1 min and 72°C for 1 min, and a final incubation at 72°C for 7 min. The reaction mixture contained 1 µl of 1:100-fold diluted cell extract, 0.2 µM of each primer, 1× PCR buffer (Bioline, London, UK), 1.5 mM MgCl_2_ and 1 unit of BiotaqTM DNA polymerase (Bioline). One µl of the resulting PCR-product was then amplified by a second PCR to prepare samples for further analysis by denaturing gradient gel electrophoresis (DGGE) using the primer set 357f-GC and 518 r [2] with the following conditions: 95°C for 5 min, followed by 10 cycles at 94°C for 30 s, 55°C for 30 s and 72°C for 30 s, 25 cycles of 92°C for 30 s, 55°C for 30 s and 72°C for 45 s, and a final incubation at 72°C for 10 min. We used the same reaction mixture as described above. A total of 151 isolates were screened by DGGE analysis [2] to determine their migration position and purity and a subset of 26 strains with distinct migration positions and yielding single bands on DGGE gels were selected for sequencing.

**Preparation of communities**

From the 26 strains isolated, six were selected for the experimental study using the following criteria: (a) growth on all three substrates (glucose, galactose and xylose) at comparable rates, (b) ability to reduce tetrazolium violet and (c) assignment to different 16S rRNA-gene-defined phylogenetic groups distinguishable by DGGE and (d) insensitivity of activity to the washing procedure used for inoculum preparation. Stock cultures of isolates were preserved in medium containing 3 mM each of glucose, xylose and galactose and 20% glycerol and stored at -80°C.

All communities were prepared using mineral salts medium 457 according to the recipe given above containing 0.01% of the redox dye tetrazolium violet (Sigma-Aldrich, St. Louis, USA).

**References**

1. Lane DL (1991) 16S/23S sequencing. In: Stackebrandt E, Goodfellow M, editors. Nucleic acid techniques in bacterial systematics. Chichester, England: Academic Press. pp. 115-175.

2. Muyzer G, Dewaal EC, Uitterlinden AG (1993) Profiling of complex microbial populations by Denaturing Gradient Gel-Electrophoresis analysis of polymerase chain reaction amplified genes coding for 16S ribosomal-RNA. Appl Environ Microbiol 59: 695-700.
