# Supplementary material for: Bacterial Biodiversity-Ecosystem Functioning Relations Are Modified by Environmental Complexity
Source: PLoS One. 2010 May 26;5(5):e10834. doi: 10.1371/journal.pone.0010834 (PMC2877076; doi:10.1371/journal.pone.0010834)
Supplement: Text S2 — Model details. Details of the linear regression models to analyse effects of species richness, species composition and substrate richness on 1) metabolic activity, 2) non-transgressive overyielding, and 3) transgressive overyielding. (0.03 MB DOC) [file pone.0010834.s004.doc]

**Models testing species richness and species composition effects on resource utilization**

**a) Species richness model**

*Model structure:*

Substrate oxidation ~ Species richness + Substrate richness + Time + Species richness × Time

*Model details:*

We used an “unstructured” variance-covariance matrix. This assumed that each variance and covariance was unique. Each mesocosm had its own variance and each pair of mesocosms had their own covariance. The gls extension allowed different levels of variance at each time point.

*Significant terms:*

2-way interaction term:

Species richness × Time (L = 127.31, d.f. = 35, p < 0.0001)

Single independent term:

Substrate richness (L = 44.83, d.f. = 12, p < 0.0001)

**b) Species composition model**

*Model structure:*

Substrate oxidation ~ Species composition + Substrate richness + Time + Species composition × Substrate richness + Species composition × Time + Substrate richness × Time + Species composition × Substrate richness × Time

*Model details:*

We used an “unstructured” variance-covariance matrix. This assumed that each variance and covariance was unique. Each mesocosm had its own variance and each pair of mesocosms had their own covariance. The gls extension allowed different levels of variance at each time point.

*Significant terms:*

3-way interaction term:

Species composition × Substrate richness × Time (L = 742.85, d.f. = 620, p < 0.001)

**Overyielding models**

1. **Species richness and substrate richness effects on non-transgressive overyielding**

*Model structure:*

Non transgressive overyielding ~ Species richness + Substrate richness + Time + Species richness × Time + Substrate richness × Time

*Model details:*

A linear regression using a gls extension with Time as a variance-covariate.

*Significant terms:*

2-way interaction terms:

Species richness × Time (L = 94.65, d.f. = 20, p < 0.0001)

Substrate richness × Time (L = 31.14, d.f. = 10, p < 0.001)

1. **Species richness and substrate composition effects on non-transgressive overyielding**

*Model structure:*

Non-transgressive overyielding ~ Species richness + Substrate composition + Time + Species richness × Time +Substrate composition × Time

*Model details:*

A linear regression using a gls extension with Time as a variance-covariate.

*Significant terms:*

2-way interaction terms:

Species richness × Time (L = 103.86, d.f. = 20, p < 0.0001)

Substrate composition × Time (L = 128.91, d.f. = 30, p < 0.0001)

**Transgressive overyielding models**

1. **Species richness and substrate richness effects on transgressive overyielding**

*Model structure:*

Transgressive overyielding ~ Species richness + Substrate richness + Time + Substrate richness × Time

*Model details:*

A linear regression using a gls extension with Time as a variance-covariate.

*Significant terms:*

2-way interaction term:

Substrate richness × Time (L = 26.08, d.f. = 10, p < 0.01)

Single independent term:

Species richness (L = 280.53, d.f. = 4, p < 0.01)

1. **Species richness and substrate composition effects on transgressive overyielding**

*Model structure:*

Transgressive overyielding ~ Species richness + Substrate composition + Time + Species richness × substrate composition + Substrate composition × Time

*Model details:*

A linear regression using a gls extension with Time as a variance-covariate.

*Significant terms:*

2-way interaction terms:

Species richness × substrate composition (L = 37.23, d.f. = 23, p < 0.0001)

Substrate composition × Time (L = 160.99, d.f. = 30, p < 0.0001)
