# Supplementary material for: Bacterial Biodiversity-Ecosystem Functioning Relations Are Modified by Environmental Complexity
Source: PLoS One. 2010 May 26;5(5):e10834. doi: 10.1371/journal.pone.0010834 (PMC2877076; doi:10.1371/journal.pone.0010834)
Supplement: Text S3 — Description of GLS extension. Brief explanation of the generalised least squares (GLS) extension for linear regression models. (0.03 MB DOC) [file pone.0010834.s005.doc]

**Brief explanation of the generalised least squares (GLS) extension for linear regression**

One of the basic assumptions of linear regression is homogeneity of variance. When this assumption is violated then the application of linear regression becomes invalid. However, as in this study, a GLS extension to the linear regression can in effect model the heterogeneity of variance in the data and therefore account for the differences in variance, allowing linear regression to be applied. Thus GLS is basically a weighted linear regression.

The GLS extension involves modelling the variance structure by incorporating variance covariates within the model framework. There are various forms that these covariates can take, and the most appropriate one must be determined using restricted maximum likelihood estimation. The residuals from the full original linear regression model, and those incorporating the different variance covariate forms are compared using AIC scores and residual plots, in order to determine the most appropriate variance-covariate structure. The details of these variance-covariates can be found in Pinheiro and Bates [1], and their application using the statistical programming language R [2] in conjunction with the library nlme [3] is clearly explained in Zuur et al. [4]. Recent ecological applications of the GLS extension can be found in Dyson et al. [5], Bulling et al [6, 7] and Godbold et al. [8, 9]. Once the most appropriate variance-covariate structure has been found, a backwards stepwise dropping procedure using maximum likelihood estimation can then be employed to determine the appropriate fixed structure (i.e. which independent variables and interactions form the minimal adequate model).

**References**6. Bulling MT, Solan M, Dyson KE, Hernandez-Milian G, Luque P, Pierce GJ, Raffaelli D, Paterson DM and & White PCL (2008) Species effects on ecosystem processes are modified by faunal responses to habitat composition. Oecologia, 158: 511-520.

7. Bulling MT, Hicks N, Murray LM, Solan M, Raffaelli D, White PCL and Paterson DM (2010) Marine biodiversity-ecosystem functions under uncertain environmental futures. Phil Trans R Soc: in press.

8. Godbold JA, Solan M, Killham K (2009) Consumer and resource diversity effects on marine macroalgal decomposition. Oikos 118: 77-86.

9. Godbold JA, Rosenberg R, Solan M (2009) Species-specific traits rather than resource partitioning mediate diversity effects on resource use. PLoS ONE 4: e7423.doi:7410.1371/journal.pone.0007423
