# Supplementary material for: Bacterial Biodiversity-Ecosystem Functioning Relations Are Modified by Environmental Complexity
Source: PLoS One. 2010 May 26;5(5):e10834. doi: 10.1371/journal.pone.0010834 (PMC2877076; doi:10.1371/journal.pone.0010834)
Supplement: Supplementary file 5 [file pone.0010834.s002.doc]

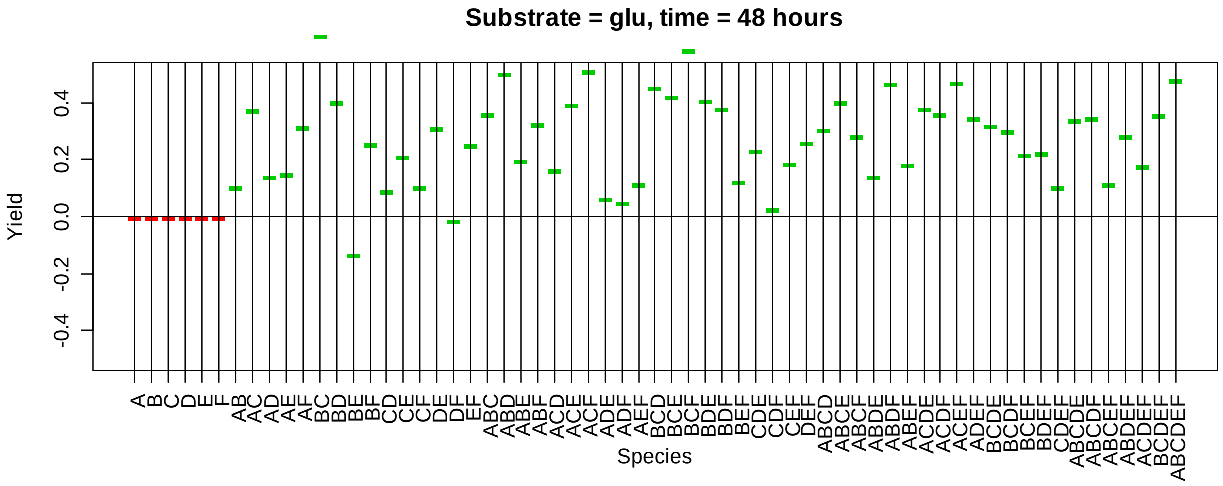
Substrate = glu, time = 48 hours
Yield
-0.4 -0.2 0.0 0.2 0.4
A B C D E F AB AC AD AE AF BC BD BE BF CD CE CF DE DF EF ABC ABD ABE ABF ACD ACE ACF ADE ADF AEF BCD BCE BCF BDE BDF BEF CDE CDF CEF DEF ABCD ABCE ABCF ABDE ABDF ABEF ACDE ACDF ACEF ADEF BCDE BCDF BCEF BDEF CDEF ABCDE ABCDF ABCEF ABDEF ACDEF BCDEF ABCDEF
Species

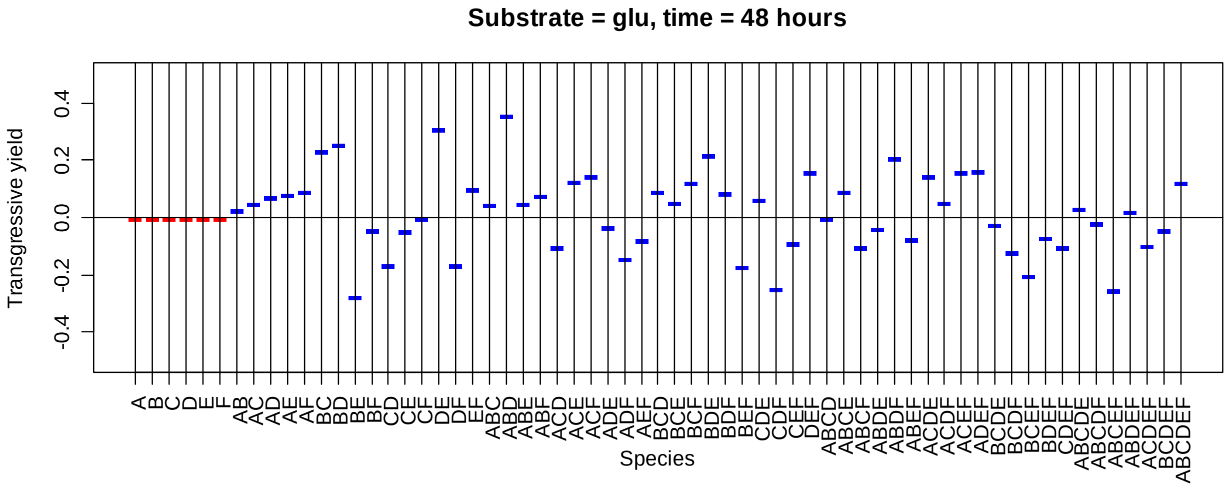
Substrate = glu, time = 48 hours
Transgressive yield
-0.4 -0.2 0.0 0.2 0.4
A B C D E F AB AC AD AE AF BC BD BE BF CD CE CF DE DF EF ABC ABD ABE ABF ACD ACE ACF ADE ADF AEF BCD BCE BCF BDE BDF BEF CDE CDF CEF DEF ABCD ABCE ABCF ABDE ABDF ABEF ACDE ACDF ACEF ADEF BCDE BCDF BCEF BDEF CDEF ABCDE ABCDF ABCEF ABDEF ACDEF BCDEF ABCDEF
Species

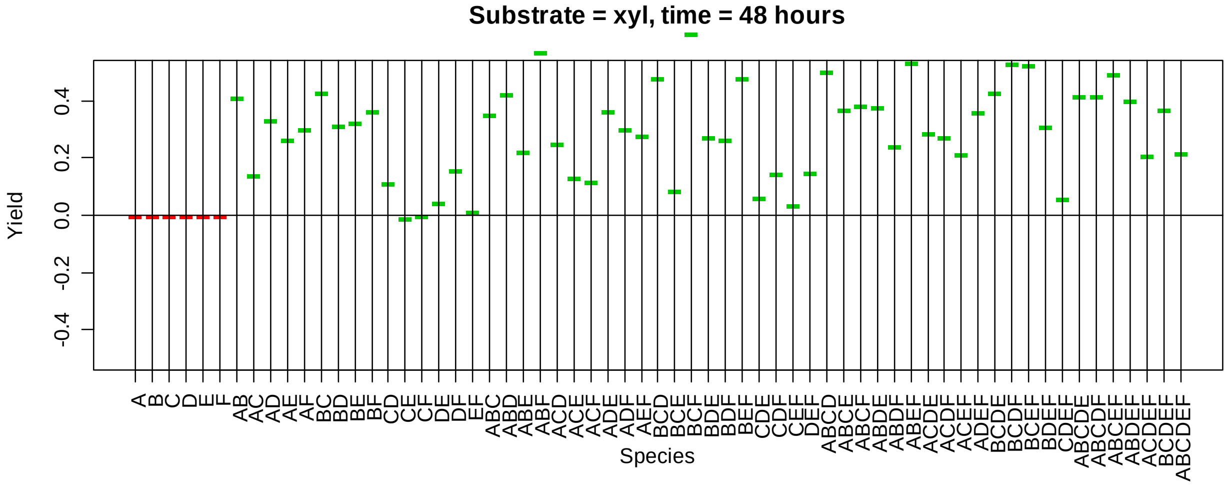
Substrate = xyl, time = 48 hours
Yield
-0.4 -0.2 0.0 0.2 0.4
A B C D E F AB AC AD AE AF BC BD BE BF CD CE CF DE DF EF ABC ABD ABE ABF ACD ACE ACF ADE ADF AEF BCD BCE BCF BDE BDF BEF CDE CDF CEF DEF ABCD ABCE ABCF ABDE ABDF ABEF ACDE ACDF ACEF ADEF BCDE BCDF BCEF BDEF CDEF ABCDE ABCDF ABCEF ABDEF ACDEF BCDEF ABCDEF
Species

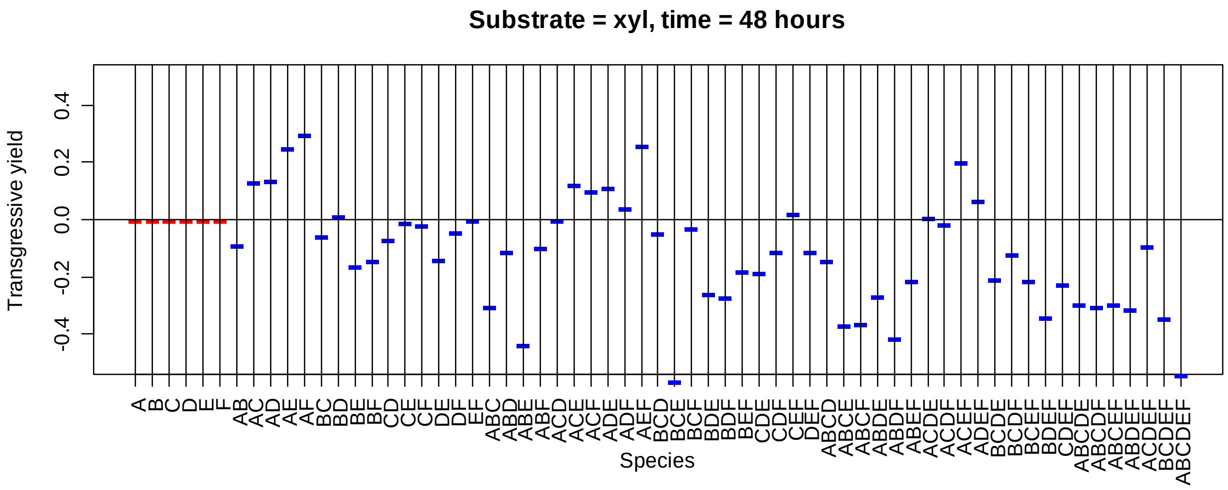
Substrate = xyl, time = 48 hours
Transgressive yield
0.4
0.2
0.0
-0.2
-0.4
A B C D E F AB AC AD AE AF BC BD BE BF CD CE CF DE DF EF ABC ABD ABE ABF ACD ACE ACF ADE ADF AEF BCD BCE BCF BDE BDF BEF CDE CDF CEF DEF ABCD ABCE ABCF ABDE ABDF ABEF ACDE ACDF ACEF ADEF BCDE BCDF BCEF BDEF CDEF ABCDE ABCDF ABCEF ABDEF ACDEF BCDEF ABCDEF
Species

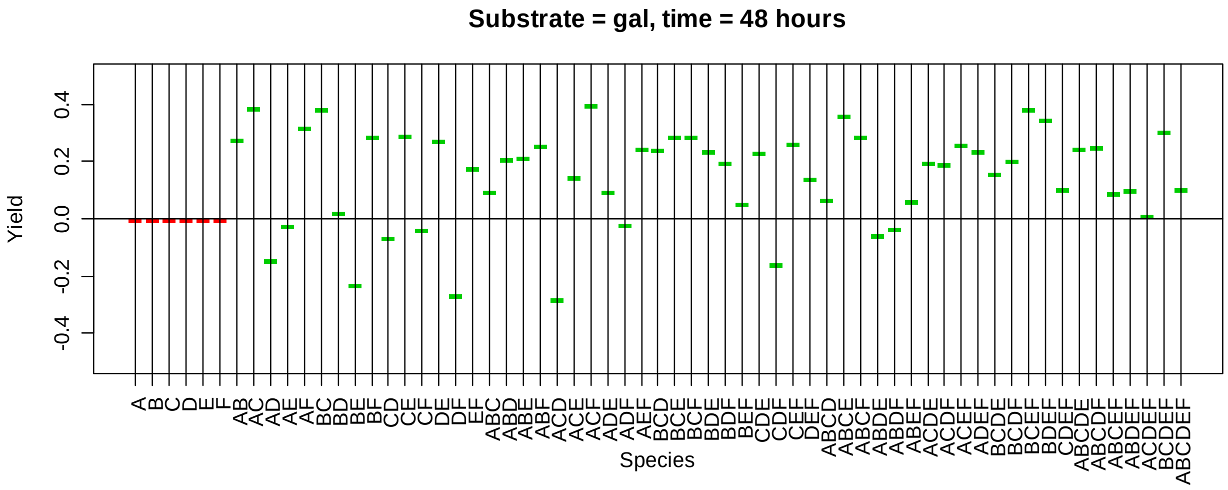
Substrate = gal, time = 48 hours
Yield
0.4
0.2
0.0
-0.2
-0.4
A B C D E F AB AC AD AE AF BC BD BE BF CD CE CF DE DF EF ABC ABD ABE ABF ACD ACE ACF ADE ADF AEF BCD BCE BCF BDE BDF BEF CDE CDF CEF DEF ABCD ABCE ABCF ABDE ABDF ABEF ACDE ACDF ACEF ADEF BCDE BCDF BCEF BDEF CDEF ABCDE ABCDF ABCEF ABDEF ACDEF BCDEF ABCDEF
Species

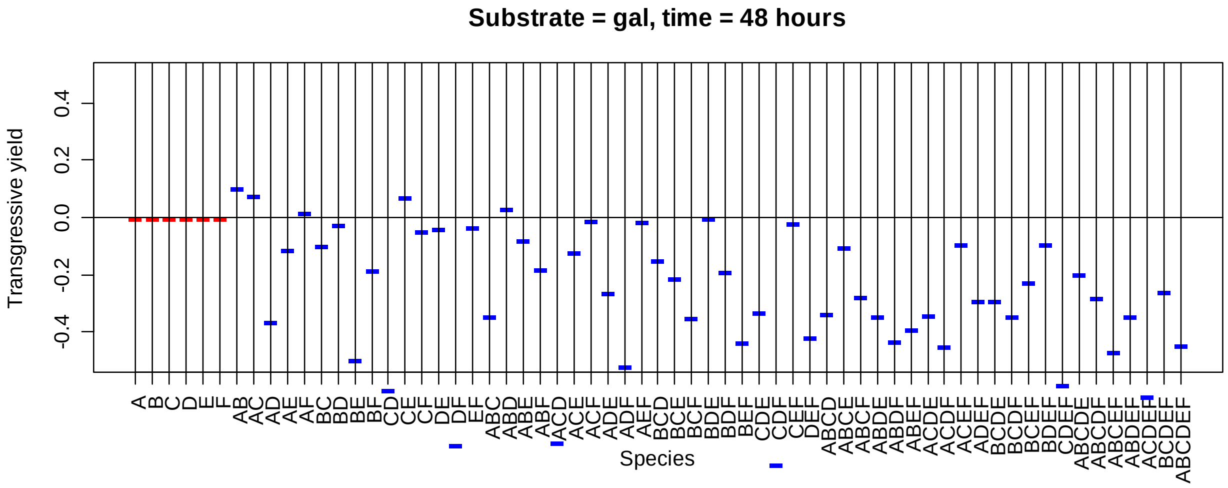
Substrate = gal, time = 48 hours
Transgressive yield
0.4
0.2
0.0
-0.2
-0.4
A B C D E F AB AC AD AE AF BC BD BE BF CD CE CF DE DF EF ABC ABD ABE ABF ACD ACE ACF ADE ADF AEF BCD BCE BCF BDE BDF BEF CDE CDF CEF DEF ABCD ABCE ABCF ABDE ABDF ABEF ACDE ACDF ACEF ADEF BCDE BCDF BCEF BDEF CDEF ABCDE ABCDF ABCEF ABDEF ACDEF BCDEF ABCDEF
Species

Substrate = gluxyl, time = 48 hours

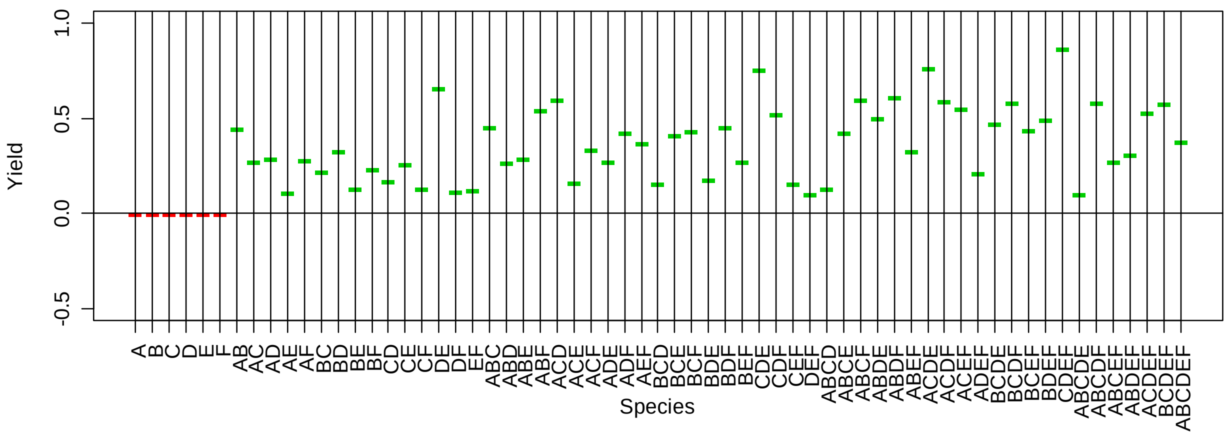

Substrate = gluxyl, time = 48 hours

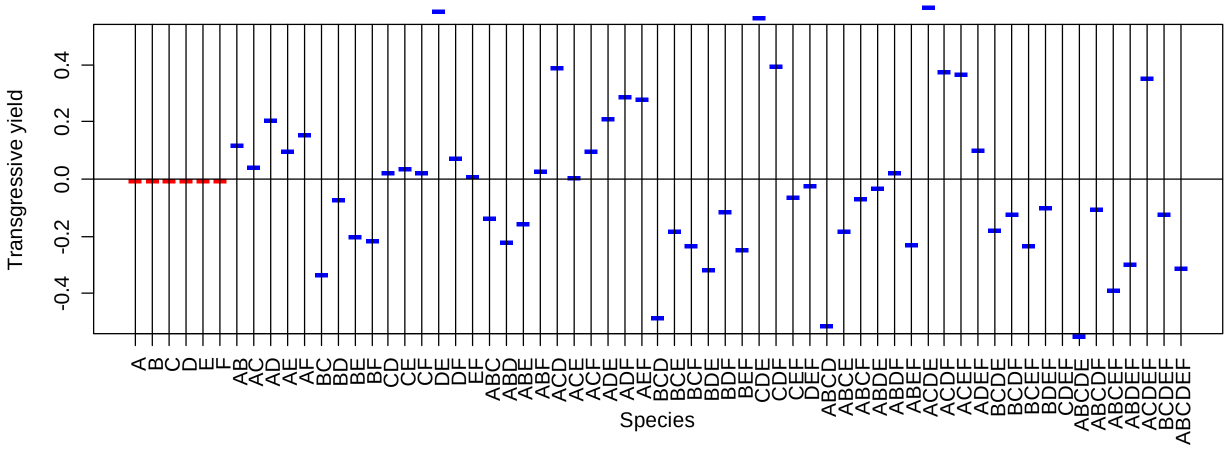

Substrate = glugal, time = 48 hours

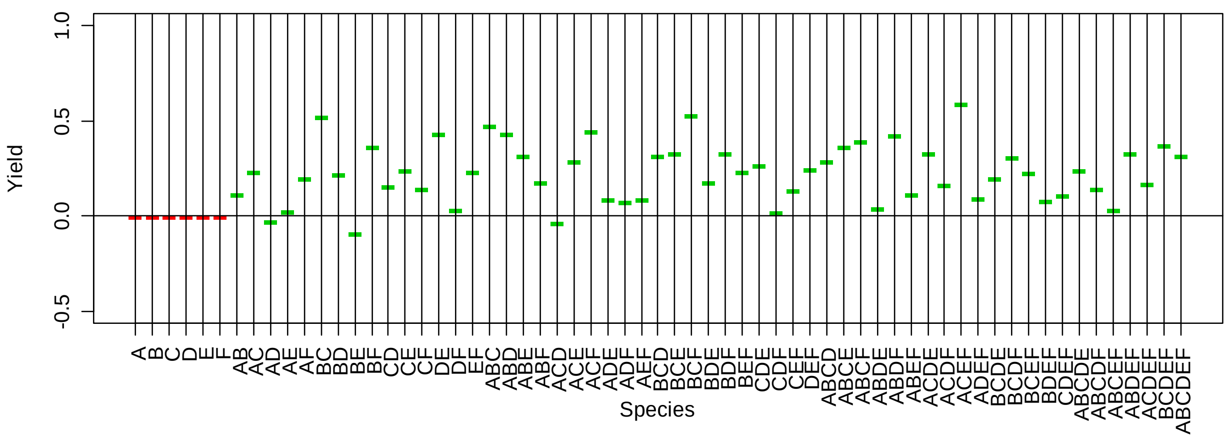

Substrate = glugal, time = 48 hours

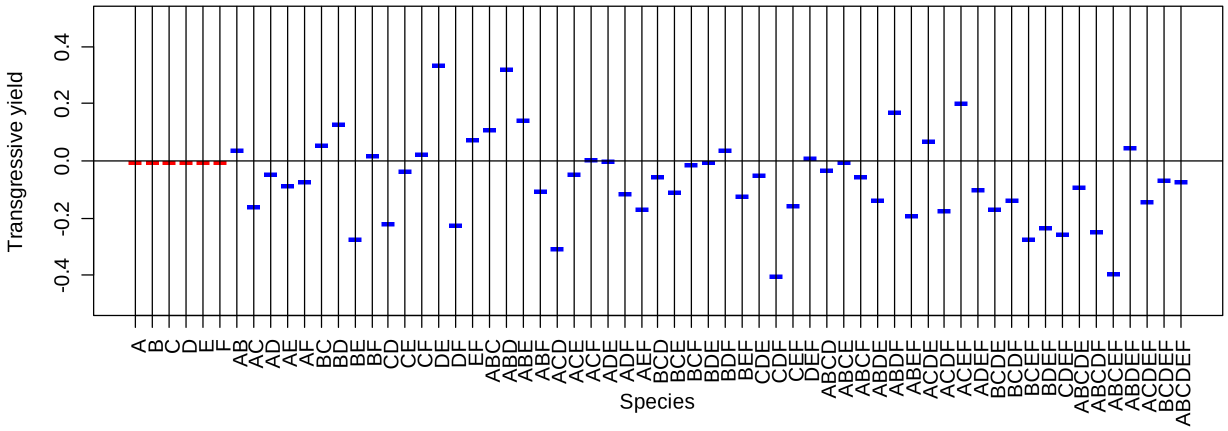

Substrate = xylgal, time = 48 hours

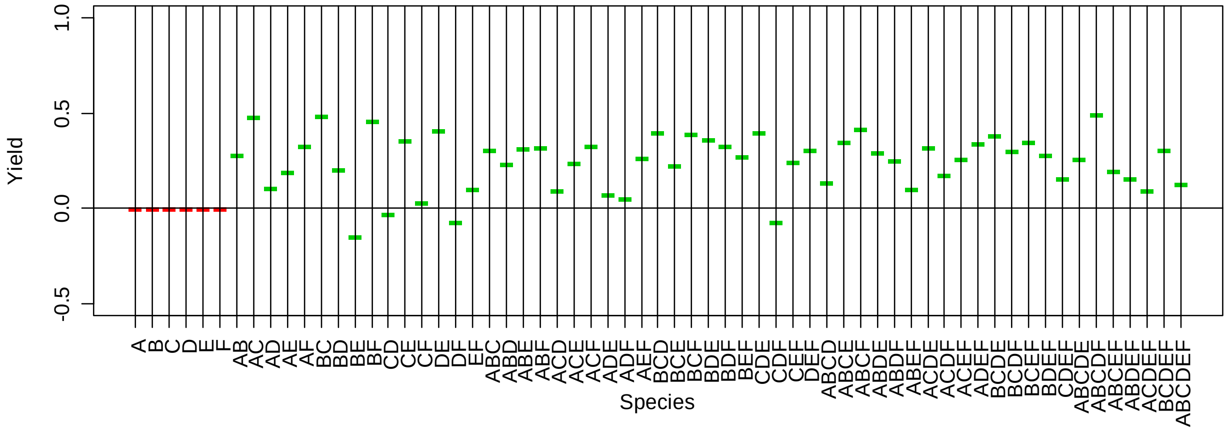

Substrate = xylgal, time = 48 hours

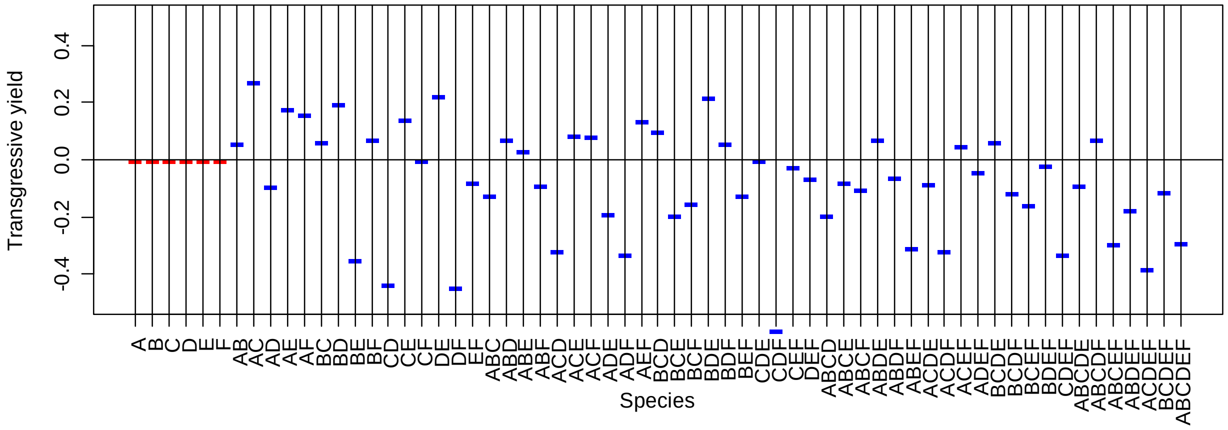

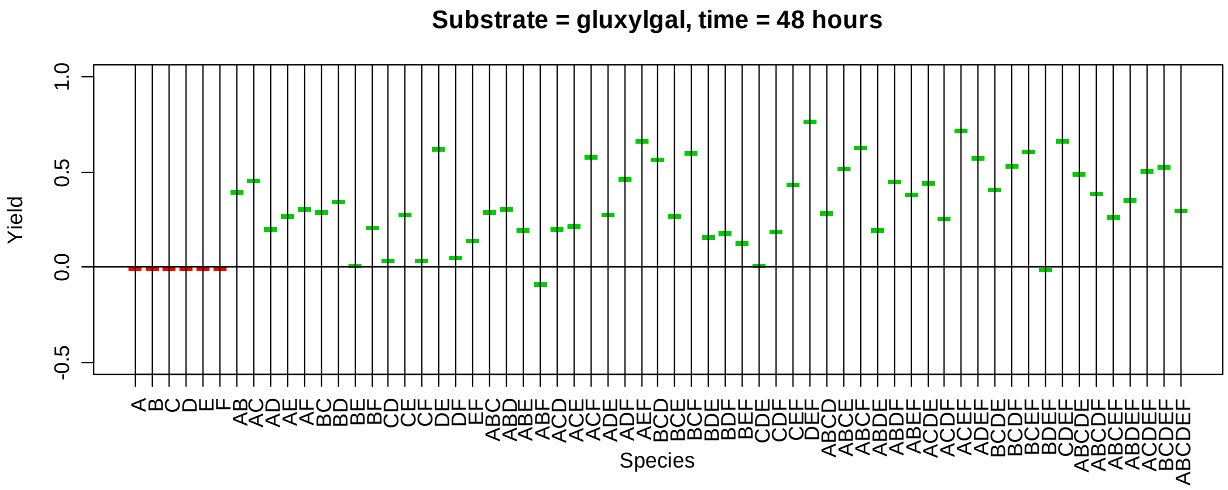
Substrate = gluxylgal, time = 48 hours
Yield
1.0
0.5
0.0
-0.5
Species
A B C D E F AB AC AD AE AF BC BD BE BF CD CE CF DE DF EF ABC ABD ABE ABF ACD ACE ACF ADE ADF AEF BCD BCE BCF BDE BDF BEF CDE CDF CEF DEF ABCD ABCE ABCF ABDE ABDF ABEF ACDE ACDF ACEF ADEF BCDE BCDF BCEF BDEF CDEF ABCDE ABCDF ABCEF ABDEF ACDEF BCDEF ABCDEF

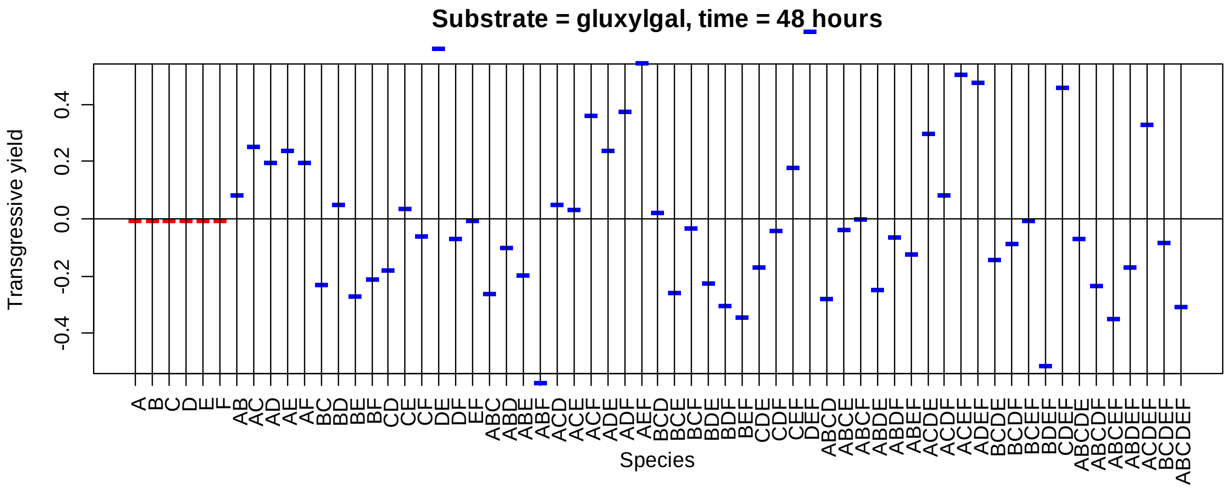
Substrate = gluxylgal, time = 48 hours
Transgressive yield
0.4
0.2
0.0
-0.2
-0.4
Species
A B C D E F AB AC AD AE AF BC BD BE BF CD CE CF DE DF EF ABC ABD ABE ABF ACD ACE ACF ADE ADF AEF BCD BCE BCF BDE BDF BEF CDE CDF CEF DEF ABCD ABCE ABCF ABDE ABDF ABEF ACDE ACDF ACEF ADEF BCDE BCDF BCEF BDEF CDEF ABCDE ABCDF ABCEF ABDEF ACDEF BCDEF ABCDEF
